# Myocardial Mechanics and Valvular and Vascular Abnormalities in Cardiac Amyloidosis

**DOI:** 10.3390/jcm13154330

**Published:** 2024-07-25

**Authors:** Attila Nemes

**Affiliations:** Department of Medicine, Albert Szent-Györgyi Medical School, University of Szeged, Semmelweis Street 8, P.O. Box 427, 6725 Szeged, Hungary; nemes.attila@med.u-szeged.hu; Tel.: +36-62-545220; Fax: +36-62-544568

**Keywords:** left ventricular, cardiac amyloidosis, myocardial, mechanics, valvular, vascular

## Abstract

Cardiac amyloidosis is an infiltrative disease primarily caused by extracellular tissue deposition of amyloid fibrils in the myocardial interstitium. The aim of the present review was to summarize findings regarding changes in myocardial mechanics, valvular abnormalities, and vascular remodeling detected in patients with cardiac amyloidosis.

## 1. Cardiac Amyloidosis

The cause of cardiac amyloidosis (CA) is the deposition of amyloid fibrils extracellularly in the myocardium and in other cardiovascular structures including the cardiac valves [1,2,3]. CA manifests as a restrictive cardiomyopathy clinically. In the presence of CA, due to the deposition of insoluble proteins, severe structural, morphological, and functional abnormalities could develop in the heart. Although currently 30 proteins are known to form a deposition extracellularly in a living tissue, literature data are available only in cases of nine proteins, which can be deposited in and around the heart. In addition to several rare and chronic inflammatory diseases (AA), monoclonal immunoglobulin light-chain (AL), hereditary transthyretin (m-ATTR), and non-mutant or wide-type transthyretin (wt-ATTR) amyloidosis are present in 98% of the cases. Although CA is a rare pathology, now it is considered to be a significantly underestimated systemic disease [1,2,3]. According to the literature, CA can be found in 43% of the autopsies in case of patients older than/equal to 75 years of age, especially in patients with left ventricular (LV) hypertrophy, heart failure (HF), and atrial fibrillation (AF) [4]. One in every ten multiple myeloma cases may have AL amyloidosis, and CA can be detected in 50–70% of subjects with AL amyloidosis [5]. In m-ATTR, since a number of genes can be involved, the exact prevalence is not known. A significant increase in the incidence (18 to 55 per 100,000 person-years) and prevalence (8 to 17 per 100,000 person-years) was found among hospitalized patients from 2000 to 2012 in a recent study [6]. In CA, the prevalence of AF varies widely according to the etiology (AL 9%, m-ATTR 11%, wt-ATTR 40%) [7]. Specifically, the prevalence of aortic stenosis (AS) is high in case of this disease [8]. Moreover, coexisting ATTR-CA was observed in every 10th elderly patient with severe AS referred for therapy [9].

## 2. Cardiovascular Imaging and Criteria

Diagnosis of CA in daily clinical practice is fraught with severe challenges. The professional guideline established by the Working Group on Myocardial and Pericardial Diseases of the European Society of Cardiology published in 2021 is a great help in diagnosing CA; it tries to clarify when to think of CA based on currently available professional and scientific results and what diagnostic procedures can help in this [1]. Based on this professional guideline, in addition to typical abnormalities found, certain extracardiac and cardiac signs found with imaging studies may draw attention to the possibility of CA. An echocardiography-derived LV wall thickness more than 12 mm verified without any other explanation is considered to be a warning sign, or a ‘red flag’. In this case, clinicians should be aware of the possibility of CA. Alternatively, these signs are common with certain types of co-occurrence [1]. 

Extracardiac red flags include (peripheral) polyneuropathy, autonomic dysfunction, bruising and discoloration of the skin, deafness, macroglossia, ruptured biceps tendon, bilateral carpal tunnel syndrome, lumbar spinal stenosis, corneal lattice dystrophy, vitreous deposits, familial accumulation, renal failure, and proteinuria [1]. 

In addition, certain specific cardiac symptoms can be considered to be warning signs, such as disproportionately elevated N-terminal-pro-B natriuretic peptide levels compared to HF (with preserved LV ejection fraction (EF)), unexplained right heart failure (with seemingly normal right ventricular (RV) and valvular function), persistent troponin level elevation, low/decreased QRS on the electrocardiogram relative to LV thickness, or a pseudo-infarction pattern (pseudo Q wave), while atrioventricular conduction disorders also raise the possibility of CA. CA may also be present in patients who have a history of hypertension but who are currently hypotonic or normotonic. Idiopathic pericardial fluid, a granular ‘sparkling’ pattern of the myocardium, a thickened RV wall and/or valve leaflet as assessed by echocardiography, and reduced LV longitudinal strain (LS) with an apical ‘sparing’ pattern measured with speckle-tracking echocardiography (STE) are also considered to be severe signs [1]. 

If cardiac magnetic resonance imaging (cMRI) examination is also available, subendocardial late gadolinium enhancement (LGE), increased extracellular volume (ECV), elevated native T1 values, and abnormal kinetics of gadolinium may be warning signs [1]. 

Other specific clinical situations potentially indicating CA include plasma cell dyscrasia, nephrotic syndrome, peripheral neuropathy, and chronic systemic inflammatory disease in patients with cardiac disease. Hypertrophy of non-dilated LV or preserved LV-EF with HF in the elderly (>65 years) may indicate the presence of hypertrophic cardiomyopathy (HCM) or severe AS [1].

There are several sets of criteria available for the diagnosis of CA, which are described in detail in the professional guideline detailed above [1]. Regarding the clinical criteria, CA may be confirmed invasively with endomyocardial biopsy (Congo red staining) or with imaging studies after extracardiac biopsy to confirm the presence of CA characteristics without other causes of LV hypertrophy by echocardiography or cMRI, for which a special set of criteria is provided. Mass spectrometry, immunohistochemistry, and immunoelectron microscopy can help determine the type of amyloid. If suspicion points in the direction of ATTR, scintigraphy is increasingly being used as a non-invasive diagnostic imaging modality, which has nearly 100% specificity to certify ATTR. In this case, ATTR-CA can be diagnosed with typical echocardiographic/cMRI if 99mTc-pyrophosphate, 99mTc-3,3-diphosphono-1,2-propanodicardioxylic acid (DPD), or 99mTc-hydromethylane diphosphonate scintigraphy confirms grade 2 or 3 myocardial uptake of the isotope, while colonial dyscrasia is excluded by serum light chain assay, urine, and serum protein electrophoresis by immunofixation. However, the presence of certain diseases (e.g., chronic kidney disease, etc.) should be taken into account [1]. 

The invasive option is endomyocardial biopsy, which directly confirms amyloid deposits using Congo red staining. This method is suitable for the verification of all CA forms; the type of amyloid can be determined by mass spectrometry, immunohistochemistry, or immunoelectron microscopy. Genetic testing allows us to determine the type of ATTR and determine whether it is m-ATTR or wt-ATTR. When confirming the hereditary form, genetic counselling and examination of the relatives are recommended as screening. After the diagnosis, in addition to treating cardiac complications and comorbidities, it is now possible to treat the disease by knowing the type of CA [1]. 

Cardiac imaging including echocardiographic and cMRI criteria for non-invasive and extracardiac biopsy-proven invasive diagnosis of CA [1]:

Echocardiography—≥12 mm LV thickness for unexplained reasons plus 1 or 2:

1. Characteristic findings (≥2 of a, b, and c have to be present):
  - Grade 2 ≤ diastolic dysfunction;
  - Decreased tissue Doppler s’, e’, and a’ wave velocities (<5 cm/s);
  - Reduced global LV-LS (<−15%).
2. Multiparametric echocardiographic score ≥ 8 points:
  - Relative LV wall thickness (interventricular septum + posterior wall)/LV end-diastolic diameter > 0.6 (3 points);
  - Doppler E/e’ > 11 (1 point);
  - Tricuspid annular plane systolic excursion ≤ 19 mm (2 points);
  - Global LV-LS absolute value ≤ −13% (1 point);
  - Systolic LS apex-to-base ratio > 2.9 (3 points).

cMRI—a and b have to be present:

- Diffuse subendocardial or transmural LGE;
- Abnormal kinetics of gadolinium;
- ECV ≥ 0.4% (strongly supportive, but not essential/diagnostic).

Due to significant improvement in cardiac imaging, new methods such as three-dimensional (3D) speckle-tracking echocardiography (STE) have joined the diagnostic options [10,11,12,13]. A virtual 3D cast of all cardiac chambers can be created non-invasively by 3D-STE, enabling simultaneous analysis of 3D volumetric parameters, deformation (strain), and rotational mechanics at the same time. In this review, findings from the ‘Motion Analysis of the heart and Great vessels bY three-dimensionAl speckle-tRacking echocardiography in Pathological cases’ (MAGYAR-Path) Study are highlighted [14,15,16,17,18,19,20]. 

The present review summarizes the most important recent findings related to CA-associated myocardial and valvular abnormalities regardless of the imaging method used, including the most important findings from the MAGYAR-Path Study [14,15,16,17,18,19,20]. Moreover, CA-associated aortic/arterial and pulmonary-artery-related abnormalities are also listed. Atrial amyloidosis, an early manifestation of CA, and the related literature is not managed separately [21]. Although AF is a frequent phenomenon in CA with a special suggested stroke-prevention anticoagulation strategy [22], AF-related abnormalities are not listed [23]. Case reports are not mentioned in this review either.

## 3. The Left Heart and the Aorta

### 3.1. Left Ventricle

#### 3.1.1. LV Structure, Volumes, Function, and Strains

**Figure 1:**
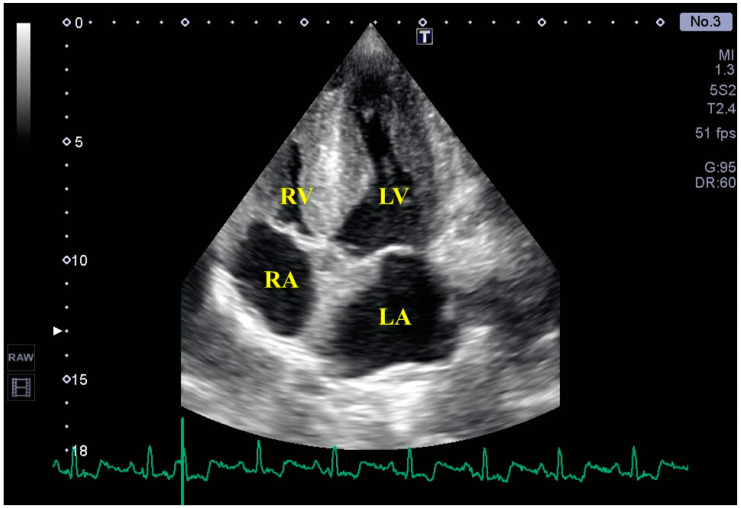
Routine two-dimensional echocardiographic image from the apical 4-chamber view of a patient with cardiac amyloidosis with biventricular hypertrophy. Abbreviations. LA = left atrium, LV = left ventricle, RA = right atrium, RV = right ventricle.

As opposed to healthy subjects [24,25], in patients with LV hypertrophy admitted for decompensation of HF, several echocardiographic features are associated with the diagnosis of CA including LV hypertrophy, decreased size of the LV cavity, relative apical sparing of strain, right atrial (RA) dilation, and alterations in right ventricular (RV) function (Figure 1). The granular ‘sparkling’ pattern of the myocardium is also a known CA feature [26]. Although LV hypertrophy is in the definition of CA, patients without an increased interventricular thickness form a relevant subgroup among those with CA [27]. Previously, although LV-EF measured during two-dimensional (2D) echocardiography was preserved in AL patients in Mayo Clinic stages II and III, evidence was demonstrated of LV systolic dysfunction, as detected by 3D echocardiography-derived LV-EF and strain analysis [28].

**Figure 2:**
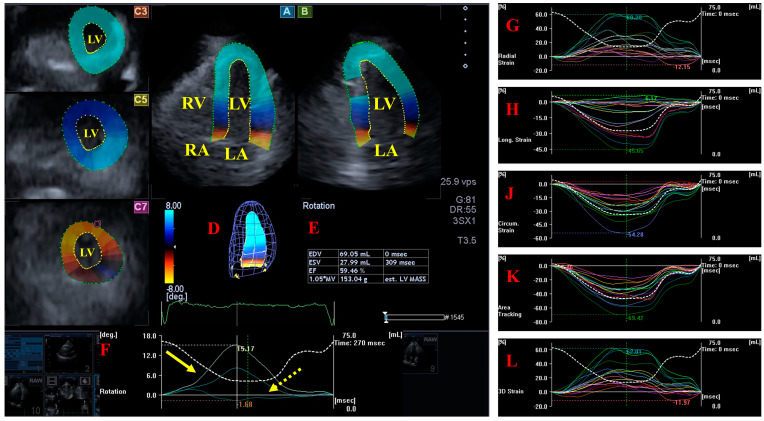
Three-dimensional (3D) speckle-tracking echocardiographic assessment of the left ventricular (LV) parameters. After 3D echocardiographic dataset acquisitions, apical 4-chamber (**A**) and 2-chamber (**B**) long-axis views and short-axis views at apical (**C3**), midventricular (**C5**), and basal (**C7**) LV levels were created using a special software. Together with a 3D virtual model of the LV (**D**), we calculated LV volumetric data and the LV ejection fraction (**E**) and apical (yellow arrow) and basal (dashed yellow arrow) LV rotations (**F**) together with curves representing time–LV global (white curve); and segmental (colored curves) radial (**G**), longitudinal (**H**), circumferential (**J**), area (**K**), and 3D (**L**) strain curves with curves representing time–LV volume changes (dashed white curve) are presented. Abbreviations. EDV = end-diastolic volume, ESV = end-systolic volume, EF = ejection fraction, MASS = LV muscle mass. LA = left atrium, LV = left ventricle, RA = right atrium, RV = right ventricle.

Systolic and diastolic LV dysfunction represented by global and segmental LV strains exists in systemic amyloidosis with preserved LV-EF [29]. In recent findings from the MAGYAR-Path Study, LV strains of all basal segments were significantly impaired in AL-CA patients. Global LV-LS of AL-CA patients was reduced as compared to global LV-LS of healthy subjects. Basal LV radial (RS) and three-dimensional (3DS) strains showed significant differences when parameters of patients with hypereosinophilic syndrome and AL-CA patients were compared [14] (Figure 2). Literature data support that a multiparametric approach with global LV-LS (and LA stiffness) may be useful for early detection of cardiac involvement associated with AL amyloidosis [30]. Endocardial LS proved to be reduced in all segments at basal, midventricular, and apical levels of LV in AL-CA patients, where the most prominent impairment could be detected at the basal level. AL-CA patients showed a significant reduction in RS in the basal LV when compared with HCM patients. HCM and AL-CA patients exhibited similar reductions in layered regional LV circumferential strain (CS) [31]. The basal LS of LV was independently associated with CA in the overall population [32]. Progressive impairment in longitudinal and basal radial LV function is a characteristic of m-ATTR-CA with unchanged global circumferential shortening [33]. wt-ATTR patients with pacemaker implantation had more impaired LV (and RV) systolic function and a higher LV mass index [34]. Resting myocardial perfusion imaging-derived strain measured in the circumferential plane may distinguish CA from other forms of LV hypertrophy [35]. cMRI-derived myocardial strain is decreased in CA, and the strain score can serve as a useful tool to identify early myocardial involvement in amyloidosis [36]. Compared to controls, a significant reduction in cMRI-derived global LV strains and LV layer-specific strains was found in CA. Global LV-RS and LV-LS, as well as subendocardial and subepicardial global LV-LS, LV-RS, and LV-CS, were all impaired in CA patientswith reduced LV-EF, when compared to patients with preserved or mid-range LV-EF [37]. Myocardial involvement in gelsolin amyloidosis is significant, but local effects are seen, mainly in the LV basal plane [38].

Although impaired global LV-LS with apical sparing is a feature of CA, low diagnostic sensitivity of relative LV apical sparing for AL-CA was demonstrated [39]. In CA patients with normal thicknesses, apical sparing was evident [40]. The relative apical sparing of LS had diagnostic value in differentiating CA and hypertensive heart disease with similar degrees and presentations of LV hypertrophy [41]. When CA patients were compared to clinically similar controls, apical sparing did not prove to be a CA-specific biomarker for accurate CA identification [42]. The apical/basal LS ratio and relative apical sparing have a diagnostic role in CA; these parameters increased gradually from carriers to patients with cardiac and neurological diseases [43].

From myocardial work indices derived from pressure–strain loop analysis, it was found that the global work index (GWI) and global constructive work were more deteriorated in ATTR-CA patients when compared with HCM and hypertensive subjects. Moreover, in the presence of preserved LV-EF, GWI had an additional discriminative value over global LV-LS alone [44]. GWI proved to be a classifier in wt-ATTR and AS versus AS [45]. In contrast, myocardial work was shown to have lower accuracy compared to the relative wall thickness or relative apical sparing in identifying ATTR-CA [46].

Regarding the differential diagnosis, in older adults, prior to transcatheter aortic valve implantation, routine four-dimensional cardiac computer tomography (CT)-derived global LV-LS and LA-LS, relative apical LS, and LV mass index showed high diagnostic performance in the detection of concomitant ATTR-CM when compared to ^99m^Tc-DPD scintigraphy [47]. Basal LV-RS was impaired in CA patients as compared to subjects with HCM, and a clear “inverse pattern” of the “physiological” gradient of basoapically decreasing LV-RS was seen in CA patients, which was reduced but still preserved in HCM. A high inverse correlation of LV-RS and cMRI-derived LGE in CA could be detected [48]. Regional myocardial strain indexed to wall thickness maybe useful in differentiating etiologies of increased LV wall thickness. Differences in myocardial deformation may be independent on wall thickness. Differences in basal strain when indexed to wall thickness in all three directions between HCM and CA are independent of LV-EF [49]. cMRI-derived global LV-LS, LV long-axis strain, global LV-RS and LV-CS were found to differentiate HCM from AL-CA with high accuracy [50]. When cMRI-derived global LV-CS, LV-LS, and LV-ECV were combined, HCM and early-stage AL-CA could be differentiated [51]. Hypertensive hypertrophy and primary CA at presentation could be differentiated by LV basal LS, and the E/e’ ratio [52]. 

From novel parameters, significantly higher myocardial stiffness was found in CA patients using both natural shear wave imaging and acoustic radiation force impulse [53]. Significantly higher end-diastolic shear wave velocities were measured in patients with CA [54]. The septal reflectivity ratio calculated as the average pixel intensity of the visible anterior septal wall divided by the average pixel intensity of the visible posterior lateral wall was found to be a reproducible and robust parameter for differentiating AL-CA from ATTR-CA [55].

#### 3.1.2. Prognostic Significance of LV Parameters

Regarding prognostic evaluations, in AL amyloidosis, global LV-LS was found to be an independent predictor of overall survival [56]. The Tei index and 2D-STE-derived global LV-LS predicted mortality in AL-CA [57,58,59]. Findings from the MAGYAR-Path Study confirmed that 3D-STE-derived global LV-LS predicted future cardiovascular events in patients with CA as well [15]. cMRI feature tracking was statistically significant in predicting death, with less impaired global LV-RS, LV-CS, and LV-LS in survivors of AL-CA [60]. LV myocardial work may have a prognostic impact in CA patients by predicting both major adverse cardiovascular events and all-cause mortality [61]. Myocardial work indices were highly correlated with markers of prognosis and were better than LV-EF (but not better than global LV-LS) in predicting mortality in CA patients [62]. GWI was demonstrated to independently predict survival in patients with wt-ATTR [45]. It was confirmed in another study in AL-CA patients that the LV myocardial work index is associated with the short-term outcome [63]. Moreover, GCW may have an additional prognostic role to global LV-LS and LV-EF in predicting HF hospitalization and all-cause mortality [64]. 

#### 3.1.3. The Role of Treatment on LV Parameters

Tafamidis treatment in patients with ATTR-CA results in a significant impairment in the standardized uptake value (SUV) retention index (serial quantitative 99mTc-DPD single photon emission computer tomography/CT for identification of cardiac amyloid burden), associated with significant benefits for global LV-LS, LV-EF, the LV cardiac index (and RV function), and cardiac biomarkers [65]. Tafamidis free acid 61 mg treatment in ATTR-CA delayed impairment of longitudinal LV function [66]. Tafamidis resulted in lesser significant deterioration in global LV-LS and the myocardial work index [67], while in another study, global LV-LS significantly improved, particularly middle and apical LS in patients with A97S ATTR-CM [68]. The decline in LV systolic and diastolic function was attenuated over 30 months by 80 mg tafamidis in patients with ATTR-CA compared to the placebo [69]. In contrast, LV function did not improve with tafamidis in another study [70]. In AL-CA, a complete hematologic response to treatment was associated with improved LV myocardial work indices. Moreover, their change was associated with improved survival [71]. In AL-CA patients who underwent chemotherapy, global LV-LS change following chemotherapy was significantly associated with overall survival as well as the response of cardiac function [72].

#### 3.1.4. LV Rotational Mechanics

Increased LV twisting and untwisting were found in patients with AL systemic amyloidosis with no evidence of cardiac involvement. In patients with evident amyloidosis and cardiac involvement, these parameters were reduced, suggesting that impaired LV relaxation induces a compensatory mechanism in the early phase of amyloidosis, which fails in more advanced stages when both twisting and untwisting rates are reduced [73]. HCM and AL-CA patients exhibited similar reductions in layered regional LV rotation and twisting [31]. In contrast, peak LV twist and untwist rates were significantly reduced in CA patients when compared with controls, while in HCM patients, increased peak LV twisting was found when compared with controls, while the peak LV untwist rate proved to be preserved [74]. LV peak basal rotation was independently associated with CA in the overall population [32]. CA and systemic hypertension both enhanced the LV twist and untwist rate before LV hypertrophy was developed. A significant LV untwisting rate peak delay was found in amyloidosis patients irrespective of the degree of infiltration of the LV [75]. In another study, m-ATTR-CA was characterized by unchanged torsion [33]. Wringing, which integrates twisting and simultaneous LV longitudinal shortening, was found to be a conditioning rotational parameter of the degree of ventricular function in patients with CA [76]. Moreover, preservation of LV-EF was found to be dependent on greater ventricular wringing [77].

In a recent study from the MAGYAR-Path Study, LV ‘rigid body rotation’ (LV-RBR), the near absence of LV twisting, seemed to be a frequent (60%) phenomenon in CA. According to our findings, 30% of the patients showed counterclockwise LV-RBR with an apico-basal LV gradient less than 3 degrees. Another 30% of the cases had counterclockwise LV-RBR with 6–10 degrees of apico-basal LV gradient. In the remaining individuals, despite the fact that LV rotational mechanics proved to be in the normal direction, their extent was different, and normo-, hypo-, and hyperrotations were observed in certain cases [16].

### 3.2. Left Atrium

**Figure 3:**
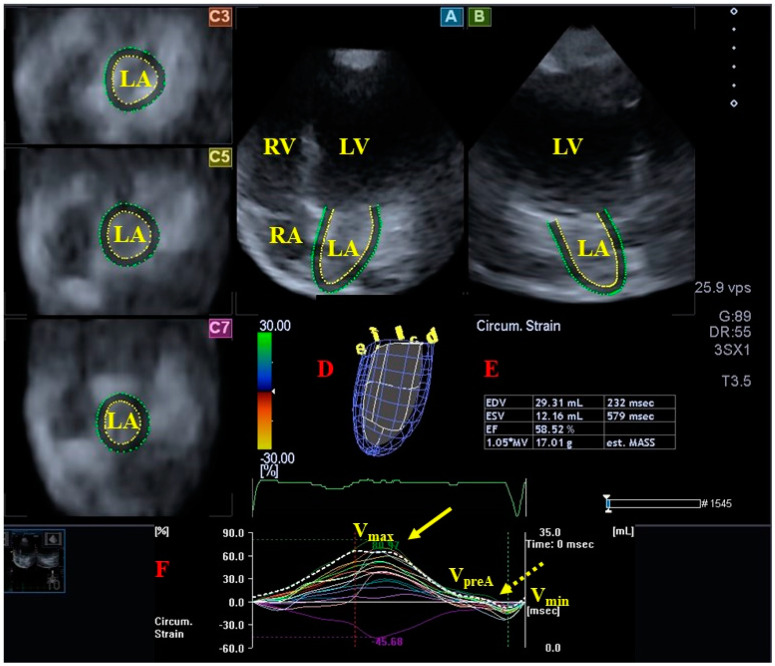
Three-dimensional (3D) speckle-tracking echocardiographic assessment of the left atrial (LA) parameters. After 3D echocardiographic dataset acquisitions, apical 4-chamber (**A**) and 2-chamber (**B**) long-axis views and short-axis views at basal (**C3**) and midatrial (**C5**) and superior (**C7**) LA levels were created using a special software. Together with a 3D virtual model of the LA (**D**), we calculated LA volumetric data (**E**) and curves representing time–LA global (white curve), and segmental (colored curves) circumferential (**F**) strains changes together with curves representing time–LA volume changes (dashed white curve) are presented. Peak LA strains are represented by a yellow arrow, while LA strains at atrial contraction are represented by a dashed yellow arrow. Abbreviations. LV = left ventricle, LA = left atrium, RV = right ventricle, RA = right atrium, EDV = end-diastolic volume, ESV = end-systolic volume, EF = ejection fraction, MASS = LA muscle mass, Vmax = minimum end-systolic LA volume, VpreA = early diastolic LA volume before atrial contraction, Vmin = end-diastolic minimum LA volume.

As opposed to what can be seen in healthy conditions [78,79,80], enlargement of the LA is a frequent finding in CA, which is related to diastolic dysfunction of the LV. In addition, atrial myopathy can occur partly due to amyloid infiltration of the LA. Functionally, increased LA stiffness and reduced LA contractility can be manifested [81]. In the context of hypertrophic phenocopies, an increased thickness of the interatrial septum together with crista terminalis and mitro-aortic lamina are suggested to have the potential to diagnose ATTR-CA [82]. In the MAGYAR-Path Study, when LA parameters of HCM and AL-CA patients were compared by 3DSTE, different patterns of LA functional characteristics were found. Significantly increased LA volumes could be detected in both disorders as compared with controls. Only the active LA emptying fraction was decreased in AL-CA patients, while reduced (peak) reservoir global LA-CS, LA-LS, and LA-AS and global LA-AS at atrial contraction were found in AL-CA patients when compared with controls. There were differences between active total and active atrial stroke volumes and certain LA strains between HCM and AL-CA patients [17] (Figure 3). Impairment of LA function was more severe in high-risk as compared to low-to-moderate-risk AL-CA patients. LA strain and the LA emptying fraction were closely associated with LV-ECV in AL-CA patients and exhibited a good capability to differentiate AL-CA patients [83]. The LA reservoir strain and peak LA-CS and LA-LS were markedly lower in ATTR-CA as compared to AL-CA [84,85]. Phasic LA and right heart strains showed the highest diagnostic accuracy to distinguish CA and Fabry disease in another study [86]. In AL amyloidosis, a multiparametric imaging approach with global LV-LS and LA stiffness was found to be helpful for detecting early cardiac involvement [30]. Peak LA strain had the strongest association with survival in CA [87]. Lower LA strain independently predicted mortality in AL-CA [88]. Moreover, LA reservoir strain was an independent predictor of new-onset AF in AL-CA patients [89]. In ATTR-CA with an increased LV wall thickness, abnormal LA myocardial function was found, irrespective of the atrial cavity size, and the LA strain rate was found to be a strong predictor of atrial arrhythmic events [90]. Tafamidis treatment delayed deterioration of LA longitudinal function in ATTR-CA [66]. In another study, tafamidis treatment improved LA function in wt-ATTR-CA patients being in sinus rhythm, while LA function was not improved in AF patients [70].

### 3.3. Mitral Valve

**Figure 4:**
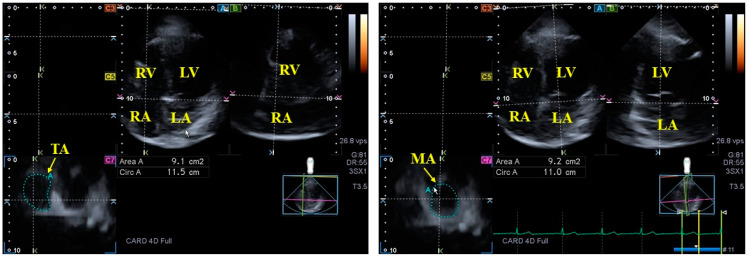
Three-dimensional (3D) speckle-tracking echocardiography-derived two-dimensionally projected views of the tricuspid (TA) (**left panel**) and mitral annuli (MA) (**right panel**). After 3D echocardiographic dataset acquisitions, following adjustments on apical four-chamber (**A**) and two-chamber (**B**) long-axis views, TA/MA-optimized images are presented in C7 cross-sectional views (**C7**). The yellow arrows represent the TA/MA plane. Abbreviations: LA = left atrium, LV = left ventricle, RA = right atrium, RV = right ventricle, Area = TA/MA area, Circ = TA/MA perimeter.

There are special mitral valve (MV) abnormalities in CA as compared to healthy subjects [91,92,93]. MV thickening was present in 75% of CA patients, and thickening of the MV and aortic valve (AV) together was also common [94]. To differentiate CA and Fabry disease (FD), MV thickness seems to be a useful parameter. Moreover, hypertrophy of the mitral papillary muscle (PM) is a typical sign of FD according to recommendations [95]. In a recent study, a significantly increased PM/LV ratio was found in FD compared to CA [96]. On the contrary, hypertrophy of the PM was less pronounced in FD as compared to CA patients in another study [97]. The histopathological findings for amyloid correlate with echocardiographic imaging of thickened, shortened, and restricted valve leaflets as well as prominent and stiff PM and chordae tendinea [94,95,96,97,98,99]. There are conflicting results regarding mitral regurgitation (MR) in CA. MR was found to be present in 62% [100] and 20.8% of the cases [97]. Atrial functional MR followed by primary infiltrative MR were the most common etiologies [101]. In other studies, equal/larger than moderate MV regurgitation was present in appr. 15% [97] and 18% [102] of CA patients. In a paper from the MAGYAR-Path Study, CA proved to be associated with dilated end-systolic and end-diastolic mitral annular (MA) dimensions and functional impairment, as assessed using 3D-STE [18] (Figure 4). ATTR-CA patients have a prominent impairment of mitral valve structure and function and higher score values [103]. AL amyloidosis patients frequently have left heart valve (mitral and aortic) thickening, which is associated with LV systolic and diastolic function, a worse functional class, and a more advanced stage of the disease. In addition, MV and AV thickening appears to be a powerful marker of all-cause mortality [104].

### 3.4. Aortic Valve

AS and MR/tricuspid regurgitation (TR) are the most common forms of valvular diseases in CA. Isolated deposition of aortic valve amyloid is common in the context of AS, but its potential clinical relevance does not appear to be important. In contrast, amyloid deposition within AV leaflets could have a role in the pathophysiology of AS. Increased local inflammation and high mechanical stress may play a significant role in the amyloidogenic process and promote the accumulation of fibrils in stenosing valves [105,106]. The diagnosis of CA and AS is challenging due to the fact that they share similar risk factors, disease mechanisms, and patterns of remodeling [107]. Equal to/larger than moderate aortic valve regurgitation was present in 13% of CA patients [97], while AS could be detected in 8% of CA patients [102]. The moderate-to-severe AS prevalence was found to be 9% in CA patients, and the majority had AL amyloidosis [108]. Moderate–severe AS prevalence at the wt-ATTR time of diagnosis was 10.5%, where most of the patients were subclassified as a low-flow, low-gradient, severe AS group [109]. The co-prevalence of AS and CA was only 4.9%, which proved to be lower than expected in another study [110]. According to literature data, the prevalence of overall pooled ATTR-CA-AS was 13.3% in AS patients who underwent transcatheter aortic valve replacement [111].

### 3.5. Aorta

Although significant dilation of aortic dimensions was not found, CA was found to be associated with reduced pulsatile change in aortic diameter and aortic strain and an increased aortic stiffness index [112,113,114]. While endothelial function was preserved in patients with wt-ATTR-CA [115], distinct peripheral vascular properties differentiated the disease from AL-CA or HF with preserved LV-EF, associated with lower peripheral and aortic blood pressure, prolonged aortic haemodynamics, and brachial artery flow-mediated dilation in the reference-population range [116]. 

## 4. The Right Heart

### 4.1. Right Ventricle

In contrast with a healthy RV [117,118,119,120,121], a thickened RV wall is considered to be a severe sign of CA [1]. RV basal free wall LS was independently associated with CA in the overall population [32]. Global RV-LS was found to have a prognostic value in AL-CA patients, providing greater prognostic power than global LV-LS and LA reservoir strain [122]. Global RV-LS had a prognostic impact in wt-ATTR-CA patients as well [123]. The contractile reserve assessed by RV free wall LS predicted major events in patients with wt-ATTR-CA [124]. The poor outcome showed associations with diffuse RV uptake of a bone tracer with single-photon-emission computer tomography imaging in ATTR-CA patients and was an independent prognosticator at diagnosis [125]. Phasic atrial strains and the global RV strain showed the highest diagnostic accuracy in distinguishing CA and Fabry disease [86]. Tafamidis treatment reduced the SUV retention index in patients with ATTR-CM, associated with significant benefits for RV-EF [65]. In systemic AL amyloidosis, 18F-florbetapir positron emission tomography/CT identified early RV amyloid prior to RV structural and functional alterations. Increasing RV amyloid with 18F-florbetapir positron emission tomography/CT, associated with a worse RV structure and function, predicted RV dysfunction and major adverse cardiovascular events [126]. For the RV, cMRI-feature tracking-derived parameters predicted mortality, and survivors of AL-CA had less reduced global RV-LS and RV-RS [60].

### 4.2. Right Atrium

**Figure 5:**
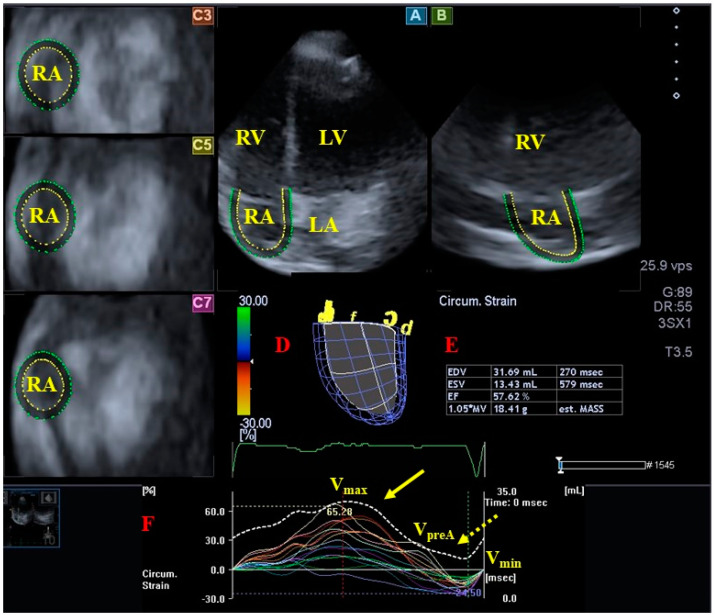
Three-dimensional (3D) speckle-tracking echocardiographic assessment of the right atrial (RA) parameters. After 3D echocardiographic dataset acquisitions, apical 4-chamber (**A**) and 2-chamber (**B**) long-axis views and short- axis views at basal (**C3**) and midatrial (**C5**) and superior (**C7**) RA levels were created using a special software. Together with a 3D virtual model of the RA (**D**), calculated RA volumetric data (**E**) and curves representing time–RA global (white curve), and segmental (colored curves) circumferential (**F**) strain changes together with a curve representing time–RA volume changes (dashed white curve) are presented. Peak RA strains are represented by a yellow arrow, while RA strains at atrial contraction are represented by a dashed yellow arrow. Abbreviations. LA = left atrium, LV = left ventricle, RA = right atrium, RV = right ventricle, EF = ejection fraction, EDV = end-diastolic volume, ESV = end-systolic volume, MASS = RA muscle mass, Vmax = minimum end-systolic RA volume, VpreA = early diastolic RA volume before atrial contraction, Vmin = end-diastolic minimum RA volume.

The RA dimensions and strain showed abnormalities in CA with a high prevalence, and abnormal values were associated with a worse prognosis, suggesting a special intrinsic RA atriopathy [127,128]. In recent findings from the MAGYAR-Path Study, significantly elevated RA volumes in respect to the cardiac cycle and deteriorated RA functions were demonstrated in AL-CA. The total and active RA emptying fractions were significantly decreased, while the peak global RA area strain (which combines LS and CS), together with some peak segmental RA-CS, RA-LS, and RA area strain, proved to be reduced. At atrial contraction, global RA-LS and RA area strain and some segmental RA-CS and RA area strains were impaired in AL-CA patients [19] (Figure 5). A staging system using RA strain during the reservoir phase and brain natriuretic peptide predicted the prognosis in patients with AL-CA [129]. Phasic RA (and LA) strains and global RV strain showed the highest diagnostic accuracy in distinguishing CA and FD [86]. The cMRI-derived RA strain and strain rate were found to be impaired in CA patients, supporting noninvasive differentiation between CA and HCM patients and controls [130]. Independent predictors of worsening HF in wt-ATTR during follow-up were pacemaker implantation prior to diagnosis and the RA volume index [34]. The presence of right-sided heart abnormality (RA volume index and lower TA peak velocities during systole and late diastole) on admission was associated with high 1-year mortality in AL amyloidosis patients with severe HF under updated therapeutic regimens [131]. Similar central hemodynamics of AL amyloidosis and ATTR were detected, and RA pressure was a major predictor of transplant-free survival [132].

### 4.3. Tricuspid Valve

Equal to/larger than moderate TR was present in 19% of CA patients [97,133]. Isolated TR (12.3%) and combined MR/TR (22.3%) are common and a prognosticator in CA, regardless of RV/LV function and CA etiology [101]. One of the most common CA-associated valvular abnormalities is TR (66%) [100]. In another study, moderate-to-severe TR was found to be common in CA as well (23%), with an independent prognostic significance in wt-ATTR patients but not in AL subjects [102]. It was demonstrated in the MAGYAR-Path Study that dilated end-systolic and end-diastolic TA sizes could be detected in CA patients. TA diameters, areas, and perimeters proved to be tendentiously higher in ATTR-CA patients as compared to those of AL-CA patients. TA functional properties proved to be impaired in CA patients and were more greatly reduced in patients with AL-CA [20] (Figure 4). The short-term outcome of AL-CA cases is associated with the tricuspid annular plane systolic excursion (TAPSE)/pulmonary arterial systolic pressure (PASP) ratio. A TAPSE/PASP ratio < 0.474 mmHg combined with systolic blood pressure < 100 mm Hg could identify AL-CA patients at increased risk of a poor prognosis [134]. 

## 5. Pathophysiological Background

Deposition of insoluble proteins and their infiltration of the cardiac tissue could explain the above-detailed abnormalities of cardiac chambers. In AL-CA, fibrils disrupt the architecture of the myocardium, and the pre-fibril oligomers have direct toxicity to the myocardial cells [135]. Moreover, not only the myocardium but also the valves and other cardiovascular structures can be infiltrated, causing complex alterations. Classic risk factors, such as age, diabetes mellitus, hypertension, dyslipidemia, AF, and gender, can also have effects in the remodeling of the ventricles and atria. Moreover, the effects of ventricular, atrial, valvular, and vascular abnormalities on each other should also be considered [1].

## 6. Clinical Implications

Currently, the management of CA involves a multidisciplinary approach, and cooperative work between cardiologists and oncologists is essential. A better understanding of CA-related abnormalities in myocardial mechanics, valvular structures, and the vasculature can help to treat the disease more adequately. In several cases, there is a significant diagnostic problem in differentiating/distinguishing CA from other disorders like FD, HCM, AS, hypertension, etc. Integrating several parameters such as echocardiographic-/cMRI-/other imaging method-derived structural and functional features could theoretically improve the differential diagnostic work-up, as demonstrated by early findings. Moreover, advanced imaging techniques including LA, RA and RV strains, cMRI-derived T1 and T2 mapping, ECV quantification, and 3DSTE-derived volumetric, strain, and rotational analysis may offer alternative diagnostic opportunities, more granular prognostication, and the possibility of evaluating the efficacity of the treatment. Moreover, CA involves all cardiac chambers, and thus, chamber-specific volumetric and functional analyses and their combination may be useful to assess cardiac dysfunction [136]. In the future, it will be important to find disease-specific characteristics of high prognostic significance that can be examined and quantified with imaging studies, which will help to set up an even more accurate diagnosis.

## 7. Conclusions

CA is associated with significant myocardial, valvular, and vascular morphological and functional abnormalities with significant prognostic impacts. Subtypes of CA show differences in certain features.
